# Bioactive Compounds from *P. pertomentellum* That Regulate QS, Biofilm Formation and Virulence Factor Production of *P. aeruginosa*

**DOI:** 10.3390/molecules28176181

**Published:** 2023-08-22

**Authors:** Lida V. Hernández-Moreno, Ludy C. Pabón-Baquero, Juliet A. Prieto-Rodriguez, Oscar J. Patiño-Ladino

**Affiliations:** 1 Departamento de Química, Facultad de Ciencias, Universidad Nacional de Colombia, Sede Bogotá, Bogotá 111321, Colombia; lhernandezmo@unal.edu.co (L.V.H.-M.); ojpatinol@unal.edu.co (O.J.P.-L.); 2 Escuela de Ciencias Básicas y Aplicadas, Universidad de La Salle, Bogotá 111711, Colombia; lupabon@unisalle.edu.co; 3 Departamento de Química, Facultad de Ciencias, Pontificia Universidad Javeriana, Bogotá 110231, Colombia

**Keywords:** *Piper pertomentellum*, amides, *Pseudomonas aeruginosa*, virulence factors, biofilm, quorum sensing

## Abstract

*Pseudomonas aeruginosa* is an opportunistic pathogen responsible for many nosocomial infections. This bacterium uses Quorum Sensing (QS) to generate antimicrobial resistance (AMR) so its disruption is considered a novel approach. The current study describes the antibiofilm and QS inhibitory potential of extract and chemical components from *Piper pertomentellum*. The methodo- logy included the phytochemical study on the aerial part of the species, the determination of QS inhibition efficacy on *Chromobacterium violaceum* and the evaluation of the effect on biofilm formation and virulence factors on *P. aeruginosa*. The phytochemical study led to the isolation and identification of a new piperamide (ethyltembamide **1**), together with four known amides (tembamide acetate **2**, cepharadione B **3**, benzamide **4** and tembamide **5**). The results indicated that the ethanolic extract and some fractions reduced violacein production in *C. violaceum*, however, only the ethanolic extract caused inhibition of biofilm formation of *P. aeruginosa* on polystyrene microtiter plates. Finally, the investigation determined that molecules (**1**–**5**) inhibited the formation of biofilms (50% approximately), while compounds **2**–**4** can inhibit pyocyanin and elastase production (30–50% approximately). In this way, the study contributes to the determination of the potential of extract and chemical constituents from *P pertomentellum* to regulate the QS system in *P. aeruginosa*.

## 1. Introduction

*Pseudomonas aeruginosa* is an opportunistic pathogen and causal agent for a wide range of infections ranging from bacteremia, urinary tract, pulmonary and burn wounds. At the clinical level, it is one of the most common Gram-negative pathogens in Intensive Care Unit (ICU) infections and is responsible for high mortality [1,2,3,4]. It is also one of the main microorganisms causing nosocomial infections with a predisposition to be multiresistant in immunocompromised patients with cancer, burns, or respiratory infections [5,6,7]. The multiresistance of *P. aeruginosa* has gained importance in the last decade due to its high mortality and the costs associated with medical care, which have increased exponentially [3,8,9]. Considering that the problem of antimicrobial resistance continues to spread worldwide, it becomes necessary to implement new therapeutic alternatives. The bacterias have a communication system called quorum sensing (QS), which works by producing self-inducible molecules that occur when there is a higher population density through the activation of group behavior genes. This system regulates the expression of virulence factors, such as the production of pigments and enzymes such as elastases and proteases, which are associated with colonization, evasion of the host’s immune response and favor bacterial resistance [10,11]. The importance of controlling this system lies in decreasing the evolutionary pressure generated by bacteria when treated with antibiotics using combined therapies. In this sense, investigations have focused on the search for QS inhibitors that allow for restoring the efficacy of the antibiotic, controlling the virulence factors associated with QS and enhancing the host defense response as an advantage over invading bacteria [12,13,14,15,16].

In the search for QS inhibitors, various studies have been reported that describe the potential of extracts, essential oils and chemical constituents from plants [17,18,19,20,21,22,23,24,25,26]. This is the case of species of the genus *Piper* (Piperaceae) of which reports describe the anti-QS and anti-biofilm potential of some of its species, highlighting *P. longum*, *P. delineatum*, *P. betle*, *P. nigrum* and *P. bogotense* [20,27,28,29,30,31]. The properties of extracts of seeds of *P. nigrum*, fruits of *P. longum* and aerial part of *P. bogotense* to inhibit violacein production in *C. violaceum* have been determined [20,27,30,31]. In addition, it is found that *P. bogotense* extract and seeds of *P. nigrum* can reduce biofilm formation [31,32]. It has also been reported that *P. betle* extract could inhibit pyocyanin production and swarming motility in *P. aeruginosa* [28,33,34]. And for *P. delineatum* leaf extract has been found to have the ability to inhibit QS-associated bioluminescence in the model bacterium *Vibrio harveyi* [29]. Essential oils from *Piper* have been characterized for their potential to inhibit QS on *C. violaceum* (*P. nigrum*, *P. bogotense*, *P. brachypodom* and *P. bredemeyeri*) and biofilm formation on *P. aeruginosa* (*P. nigrum*) [27,35]. Some prenylated benzenoids, flavonoids and amides from bioactive species of this genus have been reported to have the potential to regulate the QS system that could help control multiresistant bacteria, including *P. aeruginosa* [29,30].

Among the species of this genus with biological potential is *P. pertomentellum* Trel. and Yunck., an endemic species of Colombia that has been reported at 1000–1500 m above sea level [36,37]. The ethanolic extracts from its leaves and inflorescences have been qualitatively characterized using autography on TLC, which led to the determination of their possible chemical composition (phenols, terpenoids and/or steroids) and their potential to inhibit the growth of some phytopathogenic fungi (*Fusarium oxysporum* and *Colletotrichum tamarilloi*) and the inhibition enzymes of therapeutic interest (acetylcholinesterase and pancreatic lipase) [38]. The present investigation describes the potential of extract and chemical constituents from *P. pertomentellum* as antiQS and antibiofilm on *P. aeruginosa*.

## 2. Results and Discussion

### 2.1. Phytochemical Study

To select the bioactive fractions for the phytochemical study, the ethanolic extract (EE) and the fractions obtained by CLV (DCM, AcOEt, IPA, EtOH:H_2_O 80:20) were subjected to susceptibility testing, biofilm formation (*P. aeruginosa*) and quantification in the production of violacein (*C. violaceum*). The susceptibility test results indicated that the fractions and the extract did not affect bacterial growth at any of the concentrations evaluated, except the DCM fraction, which caused inhibition at 1000 µg/mL (Table S1 in Supplementary Material). This indicates that the concentrations evaluated may not affect bacterial growth and fulfill the criteria for evaluating their potential as QS inhibitors [39,40]. In this way, it was established that the concentrations that did not affect growth were selected to evaluate the effect of the extract ethanolic and fractions (1000–3.9 µg/mL) and the fraction DCM (500–3.9 µg/mL) in the violacein production and biofilm formation assays (see Table S1 in Supplementary Material).

The potential of the extract and fractions to inhibit violacein production in *C. violaceum* and *P. aeruginosa* biofilm formation was evaluated at concentrations that do not affect bacterial growth. The results are summarized in Table 1 and suggest that only EE had an effect in reducing biofilm formation on polystyrene microtiter plates with a biofilm inhibitory concentration (BIC) of 3.9 µg/mL while EE and the DCM and EtOAc fractions inhibited violacein production with an IC_50_ between 149 and 644 µg/mL, with the extract having better activity than the fractions. It is important to note that *C. violaceum* is a biosensor used for the detection of QS inhibitor compounds because it has the ability to express a QS-controlled pigment called violacein and because it is one of the widely studied biosensor models in this field [41,42], allowing an approximation of the possible mechanism that *P. pertomentellum* substances have on QS, considering that one of the four QS systems of *P. aeruginosa* is homologous to the QS system of *C. violaceum*, which is mediated by acyl homoserine. lactones (AHL) [11,43,44].

Comparing the results with reports in the literature, it is estimated that the BIC found in this study is low, since BIC values higher than 400 µg/mL are reported for extracts of other plants [45,46]. In the case of violacein production, there are also reports of some *Piper* species that inhibit this pigment by at least 50% in the concentration range found in this study. Among the most studied species is *P. nigrum*, for which inhibitory effects on *C. violaceum* violacein production have been reported for both alcoholic seed extracts (EtOH: 40% at 500 µg/mL, MeOH: 78% at 500 µg/mL) and for fractions of different polarities [27,30,47]. It is also found that the aerial part extract of *P. bogotense* species and its fractions reduce violacein production in *C. violaceum* by more than 90% at a concentration of 1000 µg/mL [31]. Regarding the potential of *Piper* species on the biofilm of *P. aeruginosa*, it has been reported that the extract ethanolic and the DCM fraction of *P. bogotense* reduce its formation significantly with values close to 80% [31]. The most reported species is *P. betle*, whose water extract has an inhibition of 79% (3000 μg/mL) [48] and 75.35% (200 μg/mL), as well as a reduction pyocyanin of 66% (200 μg/mL) and swarming (50%), swimming (7.40%) and twitching (10.71%) with a lower concentration (25 μg/mL) [28]. According to these results, the DCM and EtOAc fractions were selected for the next step of the study.

**Figure 1:**
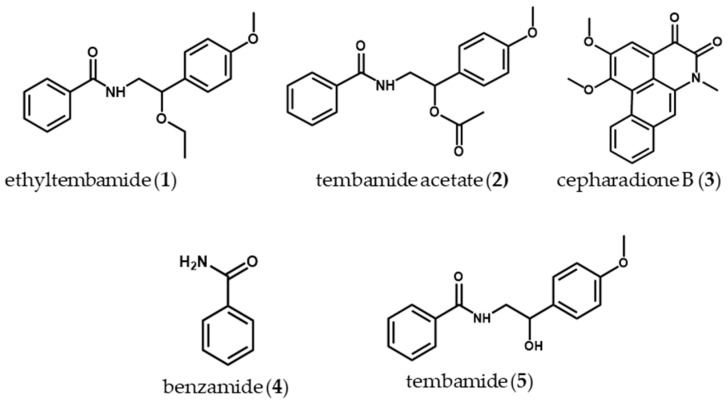
Structures of compounds isolated from *Piper pertomentellum*.

The phytochemical study performed on the DCM and EtOAc fractions from *P. pertomentellum* led to the isolation and identification of a new piperamide (ethyltembamide (**1**)), together with four known amides (tembamide acetate (**2**), cepharadione B (**3**), benzamide (**4**) and tembamide (**5**)). All compounds (Figure 1) are reported for the first time for *P. pertomentellum*; however, for other species of the genus *Piper,* the presence of **2** [49], **3** [50] and **5** [51] has been described. The structural characteristics of the compounds isolated and identified agree with the chemotaxonomy of the genus *Piper*, with amides being one of the most representative metabolite types [52,53,54,55].

Compound **1** was isolated as a white crystalline solid with m.p. 93–95 °C, which produces a dark brown coloration when sprayed on TLC with the vanillin-sulfuric acid reagent. The IR analysis for **1** (see Figure S1 in Supplementary Material) indicated the presence of amide-type functionalities (signals at 3331, 1635 and 1543 cm^−1^), aromatics (3061, 1600 and 1500 cm^−1^) and ethers (1246, 1100 and 1029 cm^−1^) [56]. According to NMR analysis (see Figures S1 and S2 in Supplementary Material), the presence of a monosubstituted aromatic ring is confirmed (^1^H signals with δ_H_ 7.78–7.74 (m, 2H), 7.54–7.48 (m, 1H), 7.44 (dd, *J* = 8.1, 6.5 Hz, 2H), together with APT signals at δ_c_ 134.7 (C), 131.5 (CH), 128.6 (2CH) and 126.9 (2CH)) and of a 1,4-disubstituted aromatic ring (δ_H_ 7.28 (d, *J* = 8.7 Hz, 2H), 6.91 (d, *J* = 8.6 Hz, 2H) and δ_c_ 159.5 (C), 131.8 (C), 127.8 (2CH) and 114.0 (2CH)). The presence of a secondary amide was also corroborated by the signal of the carbonyl group with δc 167.3 (C) and the signals of a methylene group, which via its displacement suggests being attached to a heteroatom such as nitrogen (δ_c_ 46.2 (CH_2_) and δ_H_ 3.89 (1H, ddd, *J* = 13.8, 7.8, 4.0 Hz)) [54,57]. Additionally, the presence of alkylo-xygen substituents is confirmed, corresponding to ethoxy and methoxy groups. The spectroscopic analysis performed is consistent with the structure of a tembamide-type piperamide [53,55,58]. The basic nucleus of the amide and the location of the substituents was corroborated by the analysis of two-dimensional NMR experiments (COSY, HMQC and HMBC, see Figures S3–S6 in Supplementary Material). Using high-resolution mass spectrometry analysis (HR-MS) in positive mode, the molecular formula was established as C_18_H_21_NO_3_ (m/z 296.1209 [M+H]^+^, calculated for C_18_H_21_NO_3_, 296.1281) and its formula is consistent with the result of spectroscopic analysis. Thus, compound **1** is reported for the first time and was named ethyltembamide due to its similarity with tembamide.

Compound **2** presents an NMR profile like compound **1**. The difference between their spectra is evidenced by the absence of the signals corresponding to the ethoxy group in **2** and the presence of typical signals for an acetyl group (δ_H_ 2.12 (s, 3H) and δ_c_ 21.2 (CH_3_), 170.7 (C)) (Figures S7 and S8 in Supplementary Material). This compound was identified as tembamide acetate via a comparison with data reported in the literature [59], which has been described in other species of the genus *Piper* such as *P. mollicomum* and *P. guayranum* [50,52]. Tembamide acetate has been reported to have antifungal activity (*Cladosporium cladosporoides* and *C. sphaerospermum*), antiviral activity (human immunodeficiency virus HIV-1) and antimicrobial activity (*Staphylococcus aureus*) [51,57,59].

The analysis of NMR spectra for compound **3** reveals signals typical of the 4,5-dioxoaporphine skeleton [58]. Additionally, signals were observed for two methoxy groups (δ_H_ 4.10 (d, 6H), 4.10 (d, 6H) and δ_c_ 56.6 (CH_3_), 60.6 (CH_3_)) and an N-methyl group (δ_H_ 3.83 (s,3H) and δ_c_ 29.8 (CH_3_)). Corroboration of the base nucleus and the location of the substituents was performed by HMBC analysis and by comparison with data reported in the literature [51] (Figures S9 and S10 in Supplementary Material). Thus, **3** was identified as cepharadione B, which has been isolated from different *Piper* species such as *P. augustum* and *P. longum* [50,60]. In the literature, cytotoxic, anthelmintic and antioxidant properties are reported for Cepharadione B [61,62].

Compound **4** was identified as benzamide from NMR spectroscopic analysis (Figures S11 and S12 in Supplementary Material) and by comparison with data reported in the literature [63]. This compound has not been reported in the *Piper* genus; however, it has been described for other species such as *Sarcomelicope argyrophylla*, *Houttuynia cordata* and *Haplophyllum obtusifolium* [64,65,66]. Various biological activities are known for benzamide, including antimicrobial and antibiofilm against multiresistant bacteria [64,65,66,67,68].

Compound **5** by its NMR spectra presents a similar profile to **1** and **2**, indicating that its structure corresponds to a piperamide. This compound does not present signals for the oxygenated alkyl groups located on the carbon at position **2** observed in **1** and **2**, but the signal of the methine at position **2** is maintained (δ_c_ 71.7 (CH) and δ_H_ 4.85 (^1^H, dd, *J* = 7.8, 5.1 Hz), suggesting the presence of a hydroxyl group on this position (Figures S13 and S14 in Supplementary Material). Compound **5** was identified as tembamide and has been previously isolated from *P. mollicomum* and *P. guayranum* [49,50]. Tembamide is known to have antimicrobial activity against *S. aureus* and antifungal activity against *C. cladosporoides* and *C. sphaerospermum* [51,57].

### 2.2. Effect of P. pertomentellum Compounds on Biofilm Formation and Virulence Factors of P. aeruginosa

**Figure 2:**
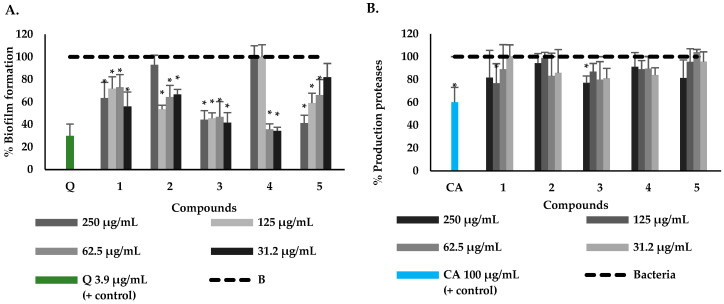

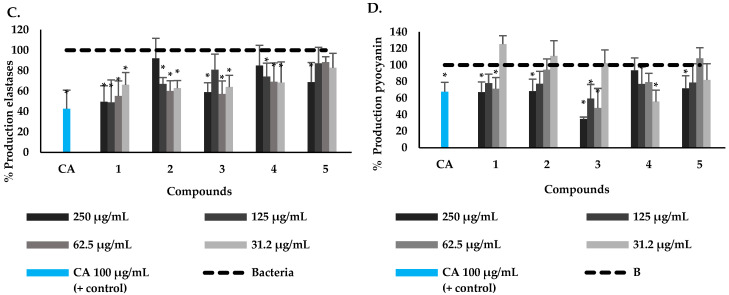
Production percentages of virulence factors of *P. aeruginosa* upon exposure to compounds isolated from *P. pertomentellum*: Biofilm (**A**), Proteases (**B**), elastases (**C**) and pyocyanin (**D**). Data are represented by the mean ± standard deviation of five independent replicates. * Indicate a significant difference according to Duncan’s test (*p* < 0.05). The inhibition controls correspond to Q = Quercetin 3.9 µg/mL for biolfilm formation and CA = cinnamic acid 100 µg/mL for virulence factors assays.

The ability of the five isolated compounds from *P. pertomentellum* to reduce biofilm formation on polystyrene microtiter plates and the production of some virulence factors of *P. aeruginosa* that are regulated by the QS system, such as elastase, protease and pyocyanin, was evaluated. It was evidenced that all compounds had a significant reduction in biofilm formation in at least two of the concentrations evaluated (Figure 2). Compound **4** showed the best activity in reducing biofilm formation at all three concentrations evaluated, with 34.2% being the lowest percentage of production at 31.2 µg/mL. In this assay, quercetin was evaluated as an inhibition control, finding that with a concentration of 3.9 µg/mL, it only allowed the formation of 27.4% of the biofilm in *P. aeruginosa* (Figure 2A) [69,70]. It should be noted that this activity has not been reported for the compounds evaluated; however, studies on certain amides that can reduce biofilm formation stand out.

Regarding the effect of *P. pertomentellum* compounds on the production of elastases, proteases and pyocyanin, it was found that compounds **1** and **3** reduced the production of three of the main virulence factors of *P. aeruginosa.* Additionally, the isolated compounds were found to have a slight reduction in protease production, finding that compounds **1** and **3** showed a significant effect with the production of 76.7% (125 µg/mL) and 77.1% (250 µg/mL), respectively (Figure 2B) and did not overcome the activity of the reference control, cinnamic acid, which produced 60.2% (100 µg/mL). Compounds **1** and **3** were the ones that reduced elastase in the greatest proportion with values of 48.8% at 125 µg/mL and 57.1% at 62.5 µg/mL, respectively, being like the control (cinnamic acid), which produced 42.6% at 100 µg/mL. Finally, in the production of pyocyanin, compounds **3** and **4** were the ones that presented the greatest effect with the production values of this pigment of 55.6% (31.2 µg/mL) and 47.9 (62.5 µg/mL), respectively. The results were better than those obtained for the control (cinnamic acid), which produced 67.6% (100 µg/mL), while compounds **2** and **5** showed a reduction in this pigment like the control at the highest concentration evaluated (250 µg/mL) with a production of 34.6%, 68.4% and 71.7%, respectively (Figure 2B–D). These results indicate the potential of natural compounds to control the virulence factors that allow the bacteria to establish infection in the host. The potential of compounds **1** and **3** in the reduction of proteases, elastases and pyocyanin is highlighted, considering that these have different functions aimed at causing damage to host tissues as the first phase of infection [71,72]. In this sense, this therapeutic strategy reduces the chances of the bacterium generating resistance and it is important to continue studying these potential molecules to identify their mechanism of action on the QS of *P. aeruginosa* [71,72]. 

For this type of compound, it has been reported that piperine and trichostachine are responsible for the quorum quenching (QQ) activity of *P. nigrum*, with inhibition values in violacein production on *C. violaceum* of 70% and 12% (50 mg/L), respectively; howe-ver, these compounds did not affect growth, biofilm and pyocyanin production by *P. aeruginosa*. The author mentioned that the QQ of both compounds is related, which could be due to their structural similarity to the Acyl-homoserine lactones (AHLs). In this way, piperamides block the QS system dependent on non-substituted AHLs due to variation of the QS signals for each system: The signal of *C. violaceum* has a non-substituted 6-carbon acyl chain whereas the signals of *P. aeruginosa* PAO1 have a non-substituted 4-carbons and beta-oxo substituted 12-carbons acyl chains [30]. In another study, it was reported that piperine decreased biofilm formation by   45% and 55% when cells were exposed to concentrations of 8 and 16 µg/mL, respectively. This result was confirmed through decreased total biofilm protein by 39% and 53% at the same concentrations and a reduction in exopolysaccharides (EPS) production of 88% at 16 µg/mL. In addition, piperine (16 µg/mL) reduced 24%, 35% and 46% of the protease secretion, rhamnolipid and pyocyanin synthesis in *P. aeruginosa*, respectively. Furthermore, the molecular simulation studies and expression of the quorum-sensing gene (*lasI*) suggested that piperine could affect the quorum-sensing network of *P. aeruginosa* [73]. In other studies, structurally diverse amide-type compounds were recently designed and synthesized that interfered with the PqsR and LasR signaling systems with reduced virulence factor production and biofilm growth in two strains of *P. aeruginosa*. Among the active compounds was *N*-cyclopentyl-5-(3-nitrophenyl)-5-oxo-pentamide, which inhibited biofilm formation by 45% (10 μM) [74]. 

## 3. Materials and Methods

### 3.1. General Experimental Procedures

For vacuum liquid chromatography (VLC), SiliaPlate ^TM^ F_254_ silica gel sized 5–20 μm (SiliCycle^®^ Inc., Quebec, QC, Canada) was used as a stationary phase. For flash chromatography (FC), SiliaFlash^®^ P 60 silica gel sized 40–63 μm (SiliCycle^®^ Inc., Quebec, QC, Canada) was used as the stationary phase. Chromatographic profiling was performed on SiliaPlate^TM^ alumina plates precoated with silica gel 60 F_254_ (SiliCycle^®^ Inc., Quebec, QC, Canada). The solvents used were technical grade and previously distilled. NMR experiments were performed on a Bruker Advance AC-400 spectrometer (Bruker ^®^, Leipzig, Germany), for ^1^H operating at 400 MHz and for APT at 100 MHz. Scalar spin-spin connectivity of ^1^H-^1^H, direct ^1^H-^13^C and long-range ^1^H-^13^C was established by 2D spectroscopic analysis with correlación protón-protón (COSY), Heteronuclear Multiple Quantum Coherence (HMQC) and Heteronuclear Multiple Bond Coherence (HMBC) experiments. The infrared (IR) spectrum was determined on an IRTracer-100 spectrometer (Shimadzu^®^, Duisburg, Germany). For high-resolution mass spectrometry analysis (HRMS), an LC-MS-TOF system (Shimadzu^®^, Duisburg, Germany) was used. The ionization method was operated with ESI in positive ion mode. Reference strains of *Pseudomonas aeruginosa* ATCC BAA-47 and *Chromobacterium violaceum* ATCC 12472, obtained commercially from the American Type Culture Collection, were used in the bioassays. For virulence factor, azocasein (Sigma-Aldrich^®^, Burlington, MA, USA) and Congo red elastin (Sigma-Aldrich^®^, Burlington, MA, USA) were used as substrates in the assays of protease and elastase, respectively. Growth of the microorganisms was performed on an orbital shaker with temperature control (Jeio tech^®^, Daejeon, Republic of Korea). Optical Density (OD) measurements were performed on a Multiskan Sky (Thermo Fischer Scientific^®^, Waltham, MA, USA).

### 3.2. Plant Material

The aerial part of *P. pertomentellum* Trel. and Yunck was collected in San Mateo town in the department of Boyacá (Colombia), at an approximate height of 2500 m above sea level. The species was determined by the biologist Ricardo Callejas and a specimen of *P. pertomentellum* Trel. and Yunck. was deposited in the Universidad de Antioquia Herbarium.

### 3.3. Extraction and Isolation of Compounds

The dried and ground aerial part of *P. pertomentellum* (200 g) was subjected to extraction by maceration at room temperature with 96% ethanol. In the process, four extractions were carried out with solvent changes every week and in each extraction the solvent was removed by using a rotary evaporator, thus obtaining 34 g of dry ethanolic extract (EE). Subsequently, 32 g of EE was fractionated by VLC with solvents of increasing polarity dichloromethane (4 g), ethyl acetate (6 g), isopropanol (10 g) and ethanol:water 80:20 (11 g). These fractions were evaluated for bacterial susceptibility, biofilm inhibition in *P. aeruginosa* and violacein production in *C. violaceum*, finding that the dichloromethane (DCM) and ethyl acetate (EtOAc) fraction had the potential to inhibit violacein production.

The DCM fraction (4 g) was subjected to flash chromatography (FC) using a mixture of hexane:EtOAc in increasing polarity (85:15 to 50:50) as the mobile phase and a total of six fractions were obtained by chromatographic monitoring (D-1 to D-6). The D-2 fraction (221 mg) was purified by successive FC with the elution systems CHCl_3_:MeOH 98:2 and hexane:acetone 70:30, obtaining **1** as a white crystalline solid (20 mg, m.p 93–95 °C). The D-3 fraction (700 mg) was subjected to successive FC with hexane:acetone 70:30 and DCM:MeOH 98:2 elution system, again obtaining compound **1** (375 mg). Subsequently, fraction D-4 (286 mg) was purified by successive FC with DCM:acetone 97:3, hexane:EtOAc 65:35 and hexane:acetone 70:30 elution systems, leading to compound **2** as a white needle-like solid (22 mg, m.p 155–157 °C). The D-5 fraction (192 mg) was subjected to successive FC purification with the hexane:EtOAc 80:20 and hexane:EtOAc 60:40 systems, yielding compound **2** (69 mg). The D-6 fraction (277 mg) was purified by successive FC with CHCl_3_ and DCM:acetone (98:2), leading to **3** as an orange-colored solid (11 mg, m.p. 266–268 °C) and **4** as a white needle-like solid (12 mg, m.p. 120–122 °C). The EtOAc fraction was subjected to flash chromatography using a 50:50 hexane:EtOAc elution system and a total of three fractions were obtained by chromatographic monitoring (A1–A3). Fraction A2 (470 mg) was purified by successive FC with the elution systems DCM:Acetone 90:10 and DCM:MeOH 95:5, yielding a white crystalline solid 5 (166 mg, m.p 154–156 °C).

Ethyltembamide (**1**): White crystalline solid; (m.p.): 93–95 °C. IR: 3331, 3061, 1635, 1543, 1600, 1500, 1246, 1100, 1029, 1029 cm^−1^. ^1^H-NMR (400 MHz, CDCl_3_): δ_H_ 7.78–7.74 (m, 2H, H-2″, H-6″), 7.54–7.48 (m, 1H, H-4″), 7.44 (dd, *J* = 8.1, 6.5 Hz, 2H, H-3″, H-5″), 7.28 (d, *J =* 8.7 Hz, 2H, H-2′ H-6′), 6.91 (d, *J* = 8.6 Hz, 2H, H-3′, H-5′), 6.60 (br. s, 1H, NH), 4.44 (dd, *J* = 8.9, 4.0 Hz, 1H, H-2), 3.89 (ddd, *J* = 13.8, 7.8, 4.0 Hz, 1H, H-1), 3.82 (s, 3H, MeOH), 3.46 (m, 1H, H-1′″), 3.41–3.31 (m, 1H, H-1), 3.41–3.31 (m, 1H, H-1′″) 1.19 (t, *J* = 7.0 Hz, 3H). APT (100 MHz, CDCl_3_): δ_C_ 167.3 (C=O), 159.5 (C-4), 134.7 (C-1″), 131.8 (C-1′), 131.5 (C-4″), 128.6 (C-3″, C-5″), 127.8 (C-2′, C-6′), 126.9 (C-2″, C-6″), 114.0 (C-3′, C-5′), 80.0 (C-2), 64.2 (C-1′″), 55.3 (CH_3_O), 46.2 (C-1), 15.3 (C-2′″). HRMS (ESI) calc. for C_18_H_21_NO_3_ [M+H]^+^: 296.1281, found: 296.1209. The spectroscopic data can be consulted in Figures S1–S6 Supplementary Material.

Tembamide acetate (**2**): White solid, melting point (m.p.): 155–157 °C. ^1^H-NMR (CDCl_3_), 400 MHz): δ_H_ 7.74 (d, *J* = 7.3 Hz, 2H, H-2″, H-6″), 7.52 (t, *J* = 7,4 Hz, 1H, H-4″), 7.46 (d, *J* = 7.8 Hz, 2H, H-3″, H-5″), 7.36 (d, *J* = 8.6 Hz, 2H, H-2′, H-6′), 6.93 (d, *J* = 8.7 Hz, 2H, H-3′, H-5′), 6.44 (br. s, 1H, NH), 5.97 (t, *J* = 6.3 Hz, 1H, H-2), 3.86 (t, *J* = 6.5 Hz, 2H, H-1), 3.83 (s, 3H, MeOH), 2.12 (s, 3H, H-2′″). APT (100 MHz, CDCl_3_): δ_C_ 170.7 (C=O), 167.4 (C=O), 159.8 (C4′), 134.3 (C-1″) 131.6 (C-3″), 129.7 (C-1′), 128.6 (C-6″), 127.9 (C-4″), 126.9 (C-2′), 114.2 (C-3′), 74.4 (C-2), 55.3 (CH_3_O), 45.0 (C-1), 21.2 (C-2″). The spectroscopic data were consistent with those reported in the literature [63]. The spectroscopic data can be consulted in Figures S7 and S8 Supplementary Material.

Cepharadione B (**3**): Yellow solid, melting point (m.p.): 267–268 °C. ^1^H-NMR (CDCl_3_), 400 MHz): δ_H_ 9.48 (t, 1H), 8.19 (s, 1H), 7.85 (t, 1H), 7.65 (t, 2H), 7.44 (s, 1H), 4.10 (d, *J* = 2.8 Hz, 6H), 3.83 (s, 3H). APT (100 MHz, CDCl_3_): δ_C_ 175.6 (C=O, C-4), 156.4 (C=O, C-5), 155.1 (C-1), 153.1 (C-2), 132.4 (C), 131.9 (C-6a), 129.2 (C-8), 128.2 (C-9), 127.8 (C-10, C-11), 127.1, 124.7 (C), 123.8 (C), 119.7 (C), 114.4 (C-7), 112.7 (C-3), 60.6 (C), 56.6 (C), 29.8 (N-Me, C-6). The spectroscopic data were consistent with those reported in the literature [70]. The spectroscopic data can be consulted in Figures S9 and S10 Supplementary Material.

Benzamide (**4**): White solid, melting point (m.p.): 119–121 °C. ^1^H-NMR (CDCl_3_), 400 MHz): δ_H_ 7.85–7.79 (m, 2H), 7.57–7.51 (m, 1H), 7.45 (dd, *J* = 8.2, 6.7 Hz, 2H), 6.04 (d, *J* = 73.12H). APT (100 MHz, CDCl_3_): δ_C_ 169.5 (C-1), 133.4 (C-2), 132.1 (C-5), 128.7 (C-4,6), 127.4 (C-3,7). The spectroscopic data were consistent with those reported in the literature [73]. The spectroscopic data can be consulted in Figures S11 and S12 Supplementary Material.

Tembamide (**5**): White solid, melting point (m.p): 155–156 °C. ^1^H-NMR (Methanol-d_4_), 400 MHz): δ_H_ 7.78 (d, *J* = 7.0 Hz, 2H, H-2″, H-6″), 7.55–7.50 (m, 1H, H-4″), 7.44 (m, 2H, H-5″), 7.34 (d, *J* = 8.7 Hz, 1H, H-2′, H-6′), 6.91 (d, *J* = 8.6 Hz, 2H, H-3′, H-5′), 4.85 (dd, *J* = 7.8, 5.1 Hz, OH), 3.78 (s, 3H, H-MeOH), 3.57 (qd, *J* = 13.5, 6.4 Hz, 2H, H-1). APT (100 MHz, Methanol-d4): δ_C_ 169.1 (C=O), 159.3 (C-4′), 134.5 (C-1″), 134.2 (C-1′), 131.2 (C-4″), 128.1 (C-3″, C-5″), 127.0 (C-2′, C-6′), 126.8 (C-2″, C-6″), 113.3 (C-3′, C-5′), 71.7 (C-2), 54.2 (CH_3_O), 47.2 (C-1). The spectroscopic data were consistent with those reported in the literature [63]. The spectroscopic data can be consulted in Figures S13 and S14 Supplementary Material.

### 3.4. Bacterial Growth Conditions

*P. aeruginosa* ATCC BAA-47 and *C. violaceum* ATCC 12472 were the reference strains selected for this study and the freezing method was used at −70 °C for long-term preservation, using 10% glycerol as cryopreservative. The working strain was obtained from the reseeding of a cryovial in Luria Bertani broth (LB) and then agar LB.

### 3.5. Evaluation of Bacterial Growth on P. aeruginosa

The broth microdilution method was performed to evaluate bacterial susceptibility following the CLSI protocol standards with some modifications [75]. In a 96-well polystyrene microtiter plate, 2 µL of each extract, fraction, or compound previously solubilized in DMSO was added. The extract and fractions were evaluated at concentrations of 1000–3.9 µg/mL and for isolated compounds 250–62.5 µg/mL as those concentrations at which the substances showed good solubility in DMSO. Then, 2 µL of the inoculum was added to obtain a concentration of 1 × 10^5^ Colony-forming Units per mL (CFU/mL) in the final well volume of 200 µL, which was completed with LB broth. The assay was carried out in triplicate and the growth control, gentamicin (2 µg/mL), pure DMSO (solvent), medium and treatments were considered. The reading was performed after 24 h in a spectrophotometer (Multiskan Sky) at OD 600 nm and the percentage of growth inhibition was calculated (Equation (1)).

(1) $$\%\;Growth\;Inhibition=\left(\frac{Abs\;control-Abs\;treatment}{Abs\;control}\right)\times 100$$

### 3.6. Biofilm Formation and Quantification Assay in P. aeruginosa

A colony was taken from a *P. aeruginosa* culture, suspended in LB broth and grown overnight (24 h) at 37 °C at 180 rpm in an incubator with an orbital shaker. In a 96-well polystyrene microtiter plate, 2 µL of the extract and fractions were added to obtain concentrations between 1000 and 3.9 µg/mL and for the isolated compounds 250–31.2 µg/mL. Then, 2 µL of the inoculum was added and the volume was supplemented with LB broth to obtain a final volume of 200 µL. Quercetin at 3.9 µg/mL was used as an inhibition control. Subsequently, it was statically incubated for 24 h. The biofilm was quantified by the crystal violet staining technique [31]. The supernatant was discarded and the plate was washed twice with PBS. It was allowed to dry completely for half an hour, then 250 µL of crystal violet (0.1% *w*/*v*) was added and allowed to act for 15 min. It was discarded and washed with PBS four times to remove excess dye and 250 µL of ethanol was added to solubilize the crystal attached to the biofilm. Readings were taken with a spectrophotometer at an OD of 570 nm and the results for extracts and fractions were expressed as the biofilm inhibitory concentration (BIC) determined as the lowest concentration that had a significant effect on biofilm formation and for compounds the percentage of biofilm formation (Equation (2)). For all assays, five replicates with two independent replicates were performed.

(2) $$\%\;Biofilm\;formation=\left(\frac{Abs\;treatment}{Abs\;control}\right)\times 100$$

### 3.7. Biosensor Quorum Sensing Assay: Quantification of Violacein from C. violaceum

The QS inhibition potential of *P. pertomentellum* extract and fractions was evaluated with the violacein quantification assay using *C. violaceum* as a bioindicator strain [76,77]. A bacterial suspension of *C. violaceum* was prepared in LB broth and adjusted to an OD of 0.4 nm. The extract and fractions were added at the same concentrations evaluated in the biofilm quantification assay. Then, 2 µL of the inoculum was added and the volume was completed with LB broth to obtain a final volume of 200 µL. The culture was incubated for 24 h at 30 °C and then centrifuged at 21,382× *g* for 10 min to separate the cells. The supernatant was discarded and 500 µL of dimethyl sulfoxide (DSMO) was added to solubilize the pigment. It was then centrifuged at 21,382× *g* for 10 min. A volume of 200 µL of the supernatant was taken and its absorbance was read at 585 nm in a 96-well plate using a microplate reader. Controls were used for growth, medium and as inhibition of thymol at 100 µg/mL. For all assays, five replicates with two independent replicates were performed and the inhibitory concentration 50 (IC_50_) was determined using GraphPad Prism 8.0.2 software with a 95% confidence level.

### 3.8. Evaluation of P. aeruginosa Virulence Factors

The potential of *P. aeruginosa* virulence factors of compounds isolated from *P. pertomentellum* extract was evaluated [78]. Initially, 5 µL of a 24 h culture of *P. aeruginosa* was added and contacted with 5 µL of different effective concentrations of the compounds in screw-capped glass tubes (250, 125 and 62.5 µg/mL) for a final volume of 500 µL and they were incubated for 24 h. Subsequently, the tubes were centrifuged at 9503× *g* for 10 min and the supernatant was used to quantify the production of proteases, elastases and pyocyanin. Cinnamic acid at 250 µg/mL was used as an inhibition control. For all assays, five replicates with two independent repetitions were performed and the results were expressed as percentage production (3).

(3) $$\%\;production=\left(\frac{Abs\;treatment}{Abs\;control}\right)\times 100$$

#### 3.8.1. Quantification of Pyocyanin Production

The extraction of pyocyanin was performed following the previously described methodology [78]. The pigment was extracted with 375 µL of chloroform and 750 µL of the supernatant. The organic layer (blue color) was acidified with 300 µL of 0.2 M HCl, which took a pink coloration. Then, 150 µL were taken and neutralized with 150 µL of a tris buffer at 200 mM, to absorbance read at 390 nm OD in microplate and pigment production was determined concerning the control.

#### 3.8.2. Quantification of Elastase Production

Elastase quantification was performed following the previous methodology [78]. From the supernatant, 25 µL was taken and 225 µL of 100 mM tris buffer pH 7.5, supplemented with 10 mg/mL elastin Congo red (ERC), was added. Subsequently, this mixture was incubated with shaking for 3 h at 37 °C. After this time, PBS buffer at pH 6.0 was added and allowed to cool for 2 min to stop the reaction. Finally, the mixture was centrifuged at 9503× *g* for 10 min to separate the insoluble ERC and an OD of 495 nm was recorded to determine elastase production.

#### 3.8.3. Quantification of Protease Production

The previous methodology for the quantification of proteases was followed [72]. First, 37.5 µL of supernatant was taken and 250 µL of a solution of 100 mM tris buffer pH 8.0 supplemented with 0.3% (*w*/*v*) azocasein was added and incubated for one hour at 37 °C statically. Next, trichloroacetic acid (TCA 10% (*w*/*v*)) was added to stop the reaction and insoluble azocasein was centrifuged at 9503× *g* for 10 min. Finally, the OD 400 nm of the supernatant was recorded to determine protease production.

### 3.9. Data Analysis

The one-way ANOVA statistical test was performed considering the assumptions of the test (normality, homogeneity of variances, independence, randomness and outliers) to determine if there were significant differences between the trials. All data showed a normal distribution according to the Shapiro test. The data that presented significant differences were subjected to an additional multiple comparison test such as Duncan for normal data, to confirm in which group the differences occurred. These analyses were performed in the R studio statistical program. All the results reported correspond to the mean of five replicates and their respective standard deviation, using a statistical significance of *p* < 0.05.

## 4. Conclusions

This paper describes for the first time the presence of five amides (**1**–**5**) in *P. pertomentellum*, highlighting the isolation of ethyltembamide **1**, which is reported for the first time in nature. In addition, this is the first report of antibacterial, antibiofilm and anti-QS activity against *P. aeruginosa* and *C. violaceum* for the ethanolic extract of *P. pertomentellum* and its isolated compounds. We identified that the chemical components of this species have the potential to attenuate the QS system of *P. aeruginosa*, highlighting the potential of **1**, **3** and **4** in the inhibition of pyocyanin and elastases and of **3** and **4** in biofilm formation.

## Figures and Tables

**Table 1:** QS inhibition potential and biofilm formation on polystyrene microtiter plates for the ethanolic extract and fractions of *P. pertomentellum*.

Ethanolic Extract and Fractions	Microorganisms	
	*C. violaceum*	*P. aeruginosa*
	* IC_50_ (95% Confidence Limit) (µg/mL)	BIC (µg/mL)
EE	149.6 (131.8–172.1)	3.9
DCM	330.9 (150.1–452.2)	NI
EtOAc	643.6 (485.1–672.3)	NI
IPA	NI	NI
EtOH:H_2_O	≥1000	NI

* IC_50_ is expressed as the mean of five replicates of an independent replicate, together with 95% confidence intervals of the mean. BIC (biofilm inhibitory concentration) corresponds to the lowest concentration that had a significant effect on biofilm formation and data are represented by the mean of five independent replicates. NI = No Inhibition.

## Data Availability

Not applicable.

## Supplementary Materials

The following supporting information can be downloaded at: https://www.mdpi.com/article/10.3390/molecules28176181/s1, Scheme S1: Isolation scheme of compounds **1** to **5** from *P. pertomentellum*; Table S1: Supplementary data on the effect of the extract and fractions on susceptibility bioassays; Figure S1: IR spectra of compound **1**; Figures S2–S14: ^1^H-NMR and APT spectra of compounds **1** to **5** and bidimensional spectra for compound **1**; Table S2: Supplementary data on the effect of the compounds of *P. pertomentellum* on susceptibility bioassays; Table S3: Formation of biofilm and production of virulence factors of *P. aeruginosa*.

## Sample Availability

Not available.
