# Supplementary material for: Bioactive Compounds from P. pertomentellum That Regulate QS, Biofilm Formation and Virulence Factor Production of P. aeruginosa
Source: Molecules. 2023 Aug 22;28(17):6181. doi: 10.3390/molecules28176181 (PMC10488431; doi:10.3390/molecules28176181)
Supplement: Supplementary file 1 [file molecules-28-06181-s001.zip › molecules-2510770-supplementary.pdf]

Supplementary material

Bioactive Compounds from *P. pertomentellum* That Regulate QS, Biofilm Formation and Virulence Factor Production of *P. aeruginosa*

Lida V. Hernández-Moreno [1], Ludy C. Pabón-Baquero [2], Juliet A. Prieto-Rodriguez [3,*] and Oscar J. Patiño-Ladino [1]

[1] Departamento de Química, Facultad de Ciencias, Universidad Nacional de Colombia, Sede Bogotá,
Bogotá 111321, Colombia; lhernandezmo@unal.edu.co

[2] Escuela de Ciencias Básicas y Aplicadas, Universidad de La Salle, Bogotá 111711, Colombia;
lupabon@unisalle.edu.co

[3] Departamento de Química, Facultad de Ciencias, Pontificia Universidad Javeriana, Bogotá 110231, Colombia; juliet.prieto@javeriana.edu.co

* Correspondence: juliet.prieto@javeriana.edu.co; Tel.: +57-6013208320 (ext. 4124)

Table of contents

1. Phytochemical study carried out on aerial part from *P. pertomentellum.*
Scheme S1. Isolation scheme of compounds **1** to **5** from *P. pertomentellum.*

2. Growth, formation biofilm of *P. aeruginosa* and violacein production of *C. violaceum* in the presence of extract and fractions of *P. pertomentellum.*

Table 1. Percentage growth, formation biofilm of *P. aeruginosa* and violacein production of *C. violaceum* in the presence of extract and fractions of *P. pertomentellum*.

3. NMR spectra from phytochemistry isolation compounds
Figure S1. IR spectra of ethytembamide (**1**).
Figure S2. ^{1}H-NMR spectra of ethyltembamide (**1**).
Figure S3. APT spectra of ethyltembamide (**1**).
Figure S4. COSY spectra of ethyltembamide (**1**).
Figure S5. HMQC spectra of ethyltembamide (**1**).
Figure S6. HMBC spectra of ethyltembamide (**1**).
Figure S7. ^{1}H-NMR spectra of tembamide acetate (**2**).
Figure S8. APT spectra of tembamide acetate (**2**).
Figure S9. ^{1}H-NMR spectra of cepharadione B (**3**).
Figure S10. APT spectra of cepharadione B (**3**).
Figure S11. ^{1}H-NMR spectra of benzamide (**4**).
Figure S12. APT spectra of benzamide (**4**).
Figure S13. ^{1}H-NMR spectra of tembamide (**5**).
Figure S14. APT spectra of tembamide (**5**).

4. Effect of compounds of *P. pertomentellum* against growth, biofilm formation and virulence factors production of *P. aeruginosa*
Table 2. Percentage growth of *P. aeruginosa* on exposure to compounds isolated from *P. pertomentellum.*

Table 3. Percentage biofilm formation and virulence factors production of *P. aeruginosa* on exposure to compounds isolated from *P. pertomentellum*.

1. Isolation compound.

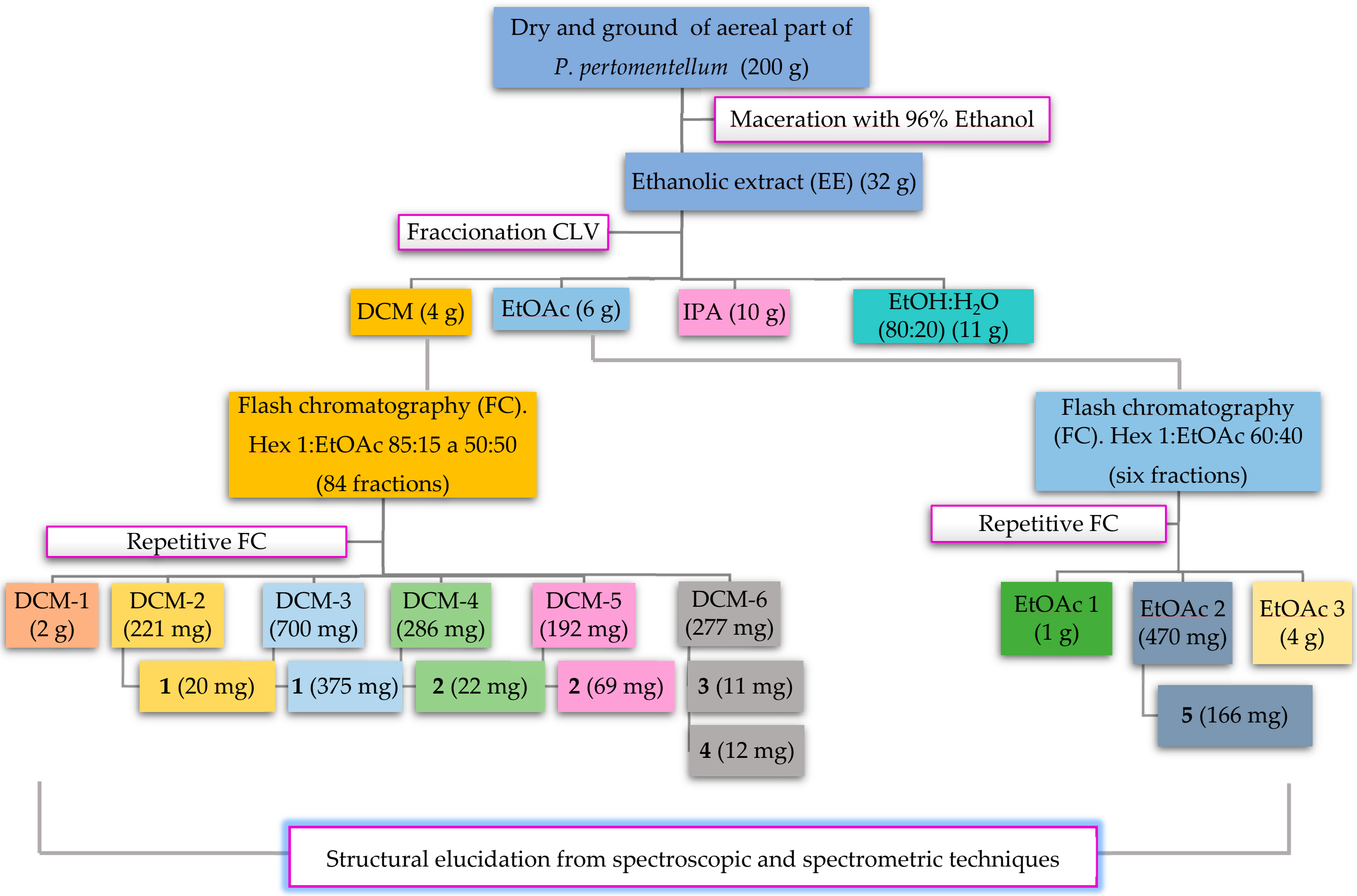

Scheme S1. Isolation scheme of compounds **1** to **5** from *P. pertomentellum*.

Table S1. Percentage growth, formation biofilm of *P. aeruginosa* and violacein production of *C. violaceum* in the presence of extract and fractions of *P. pertomentellum*.

Treatment	Concentrations μg/mL														
	1000			250			62.5			15.6			3.9		
	S	B	V	S	B	V	S	B	V	S	B	V	S	B	V
**EE**	129.1 ± 9.8	139.4 ± 19.2	9.6 ± 2.6	132.2 ± 1.3	83.4 ± 3.5	43.8 ± 4.4	122.1 ± 6.6	93.5 ± 8.6	63.9 ± 3.9	115.2 ± 4.1	77.5 ± 9.2	72.4 ± 1.7	118.5 ± 1.5	72.2 ± 17.4	81.2 ± 2.5
**DCM**	79.1 ± 1.8*	92.9 ± 7.4	10.8 ± 1.2	86.6 ± 2.6	90.1 ± 9.9	44.5 ± 8.4	95.6 ± 3.7	98.0 ± 13.8	57.6 ± 3.7	101.4 ± 2.2	92.4 ± 8.4	66.9 ± 3.3	99.8 ± 2.9	98.5 ± 18.1	86.6 ± 12.5
**AcOEt**	100.7 ± 2.7	124.3 ± 12.6	8.9 ± 1.1	103.1 ± 1.5	111.9 ± 5.1	87.5 ± 10.2	107.1 ± 1.3	101.8 ± 2.6	90.6 ± 15.7	100.1 ± 3.2	95.2 ± 16.8	100.5 ± 7.4	109 ± 2.4	84.1 ± 15.8	97.5 ± 13.5
**IPA**	100.8 ± 3.4	110.2 ± 11.0	91.9 ± 16..7	101.1 ± 2.7	131.2 ± 16.8	138.6 ± 19.2	110.7 ± 1.3	133.8 ± 12.2	130.2 ± 15.7	113.5 ± 1.7	135.2 ± 14.5	103.4 ± 16.3	111.6 ± 1.4	108.4 ± 4.5	116.5 ± 13.2
**EtOH:H_2O**	107.8 ± 4.2	162.4 ± 7.0	49.6 ± 5.1	108.5 ± 1.7	172.7 ± 19.1	51.3 ± 8.0	105.5 ± 12.1	171.8 ± 17.7	54.3 ± 11.8	103.2 ± 2.4	131.7 ± 11.9	58.3 ± 10.4	101.5 ± 1.6	104.3 ± 10.7	63.7 ± 18.9

S: growth, B: formation biofilm, V: violaceum production. Data are represented the mean ± standard deviation of five independent replicates.

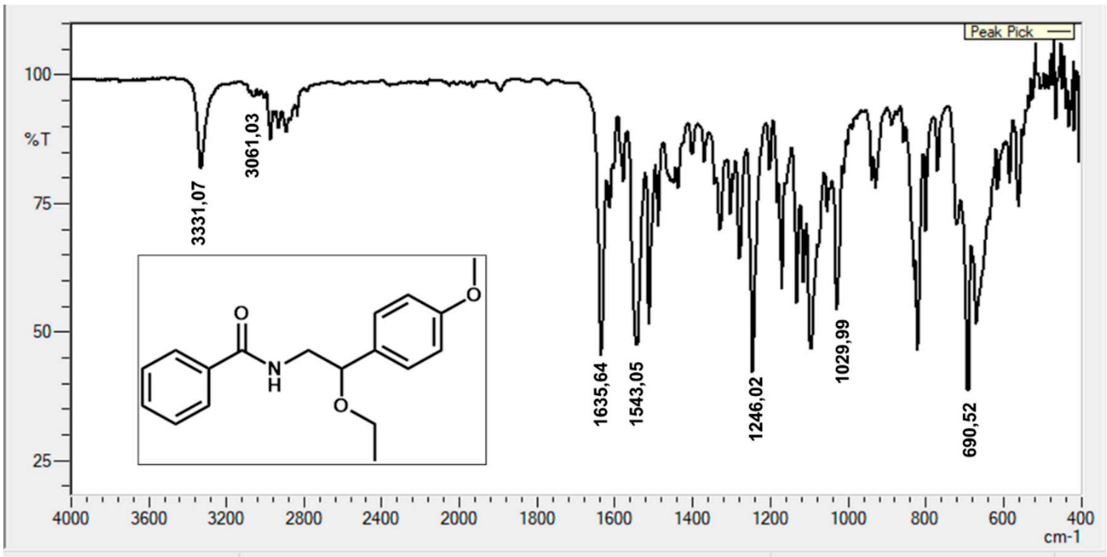

Figure S1. IR spectra of ethyltembamide (**1**).

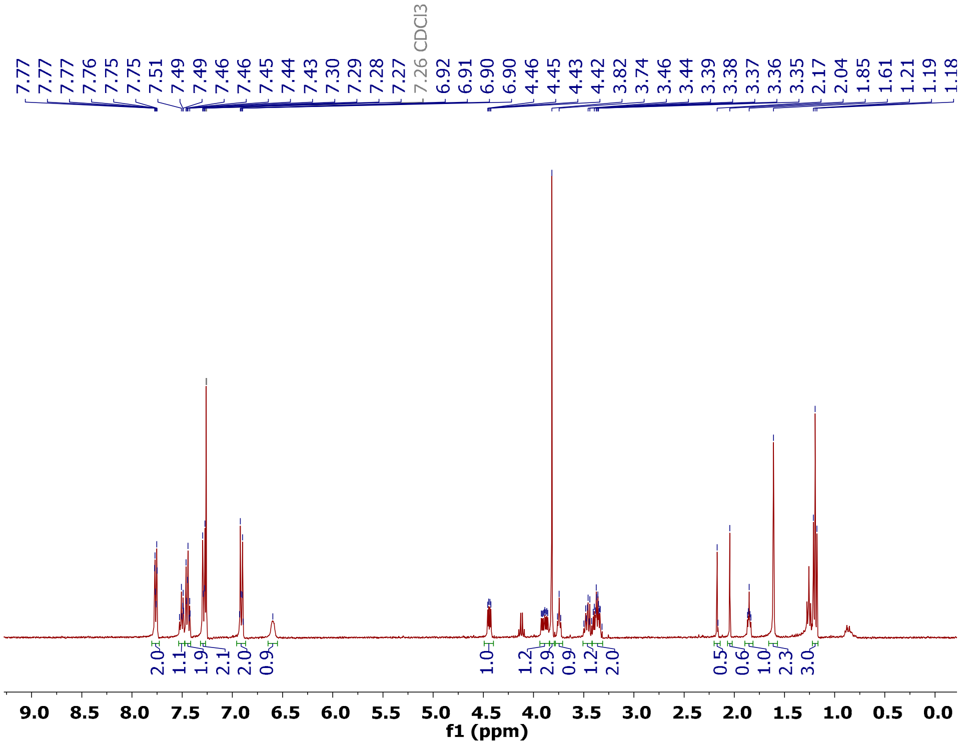

Figure S2. ^{1}H-NMR spectra of ethyltembamide (**1**).

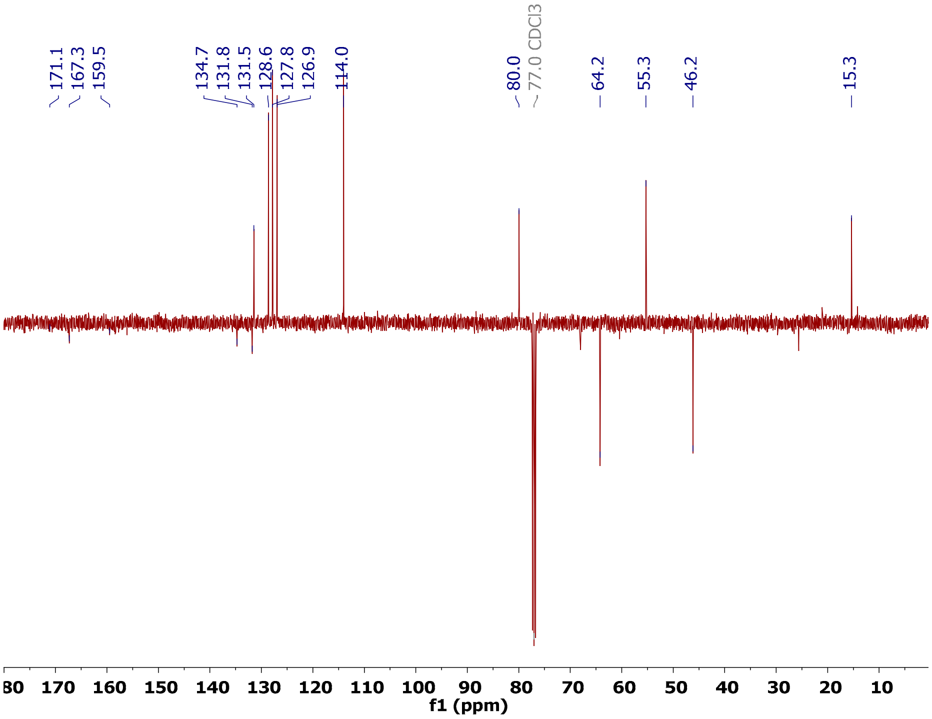

Figure S3. APT spectra of ethyltembamide (**1**).

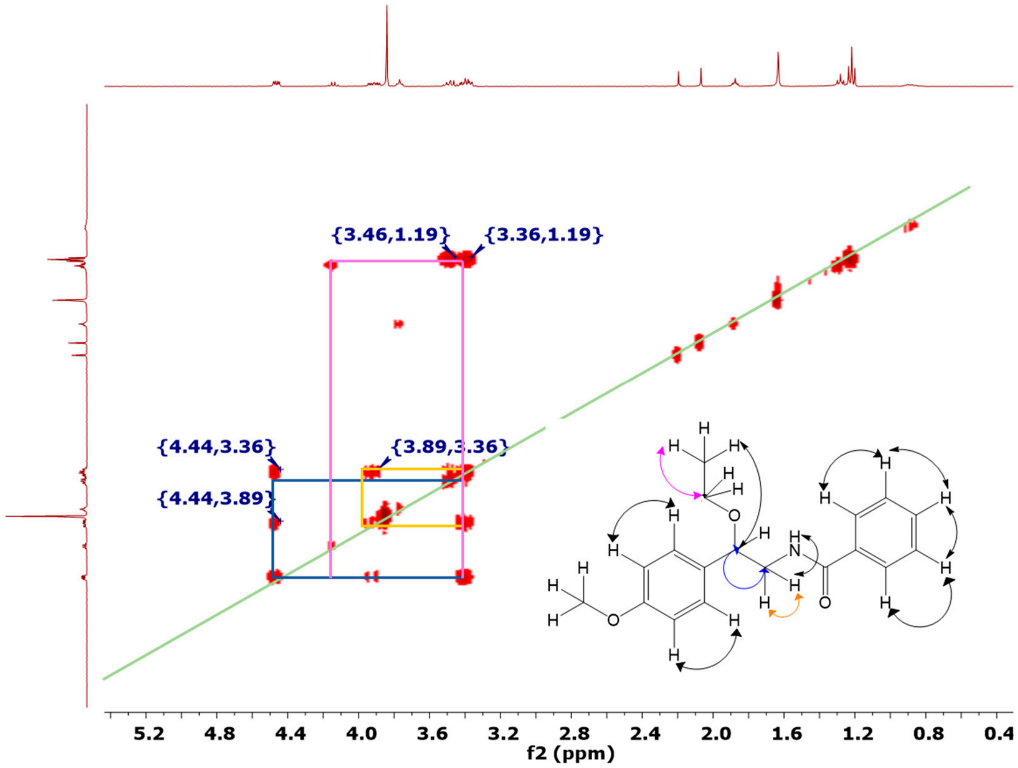

Figure S4. COSY spectra of ethyltembamide (**1**).

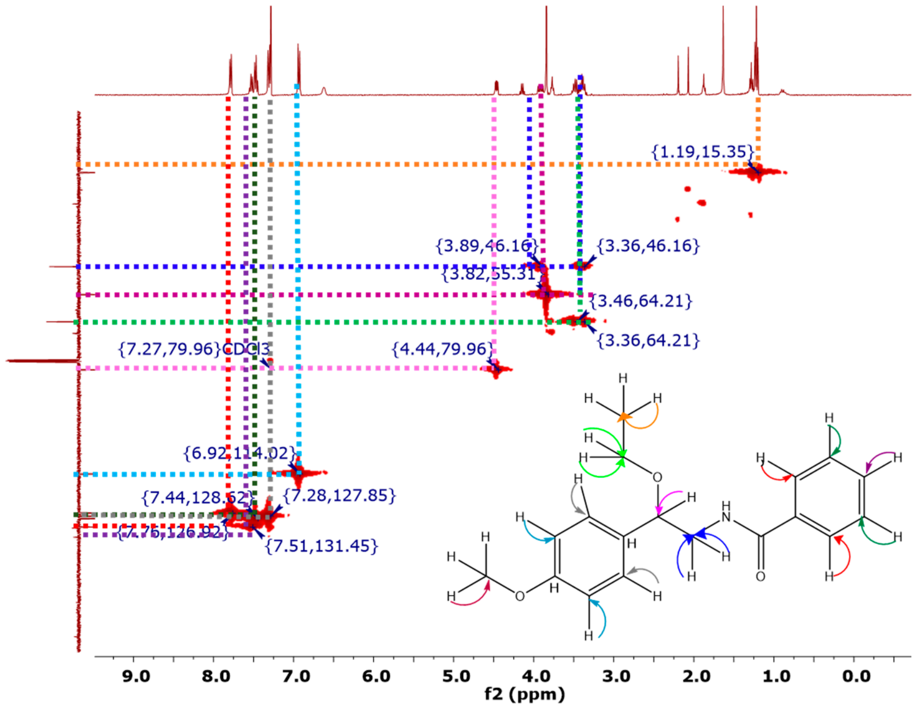

Figure S5. HMQC spectra of ethyltembamide (**1**).

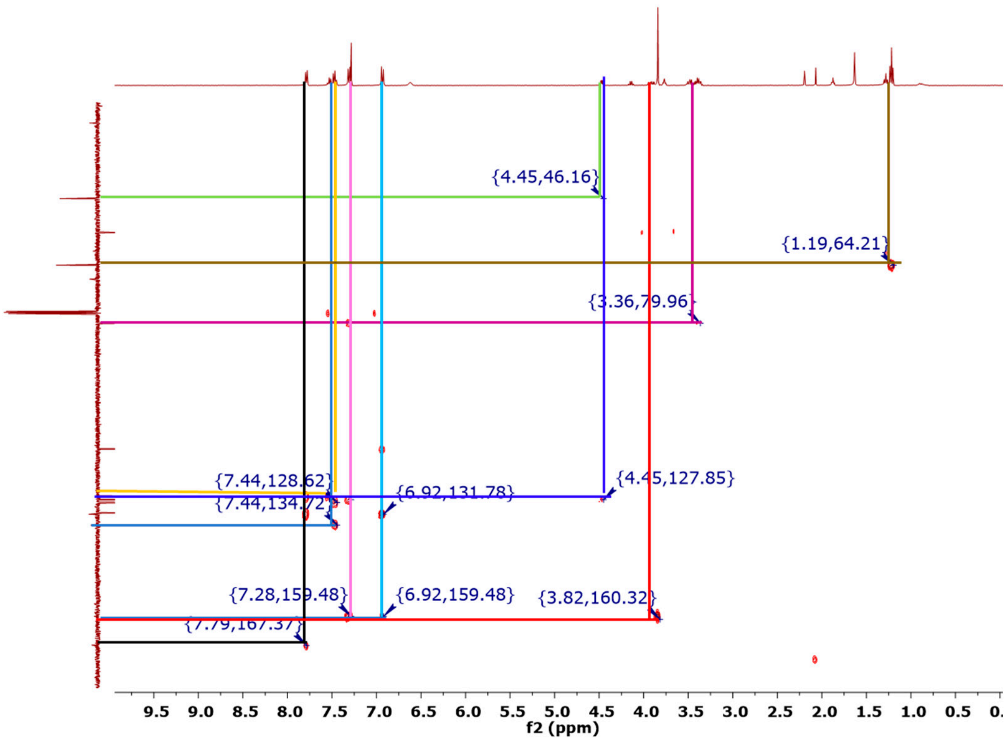

Figure S6. HMBC spectra of ethyltembamide (**1**).

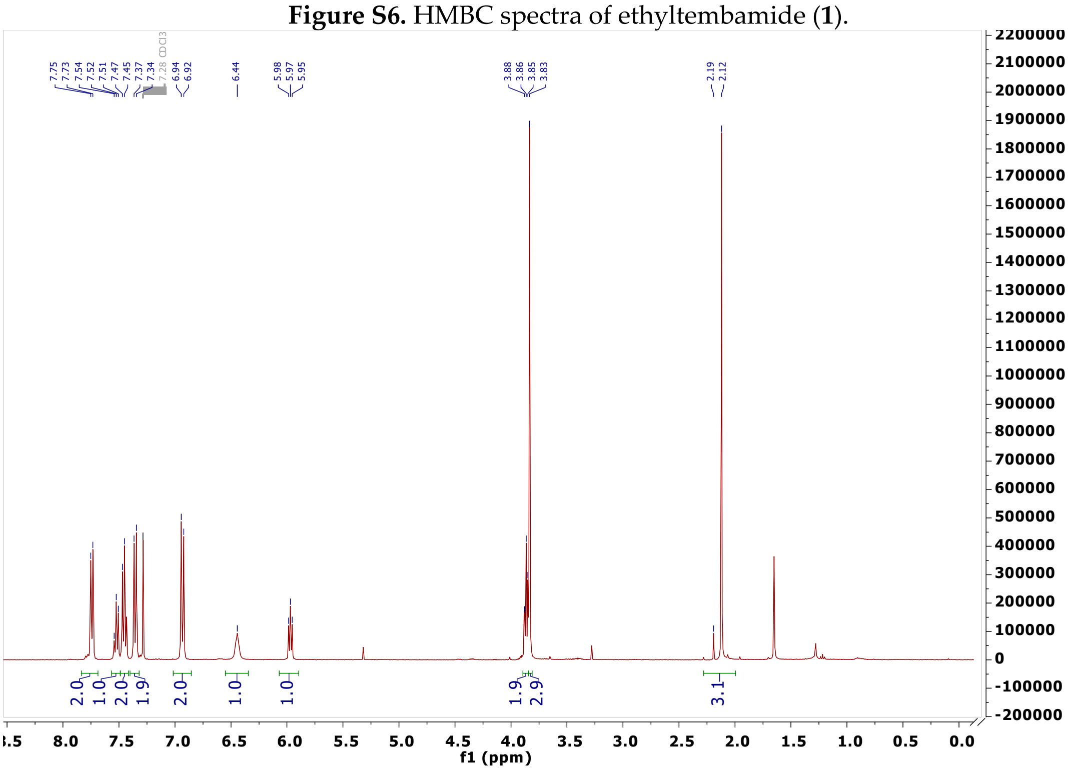

Figure S7. ¹H-NMR spectra of tembamide acetate (**2**).

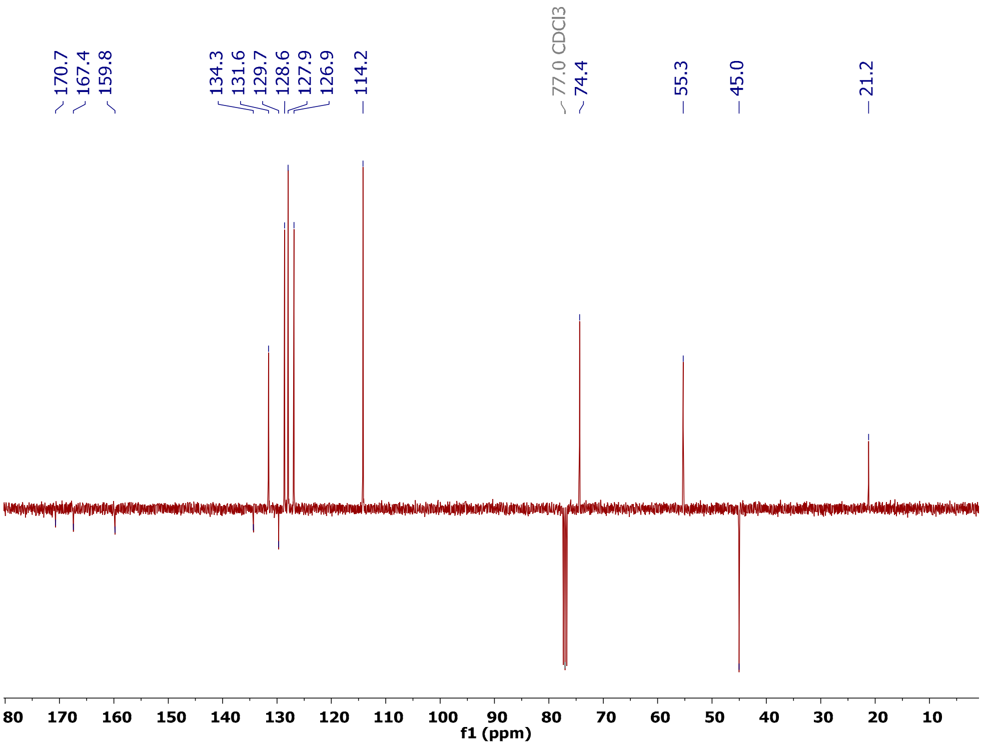

Figure S8. APT spectra of tembamide acetate (**2**).

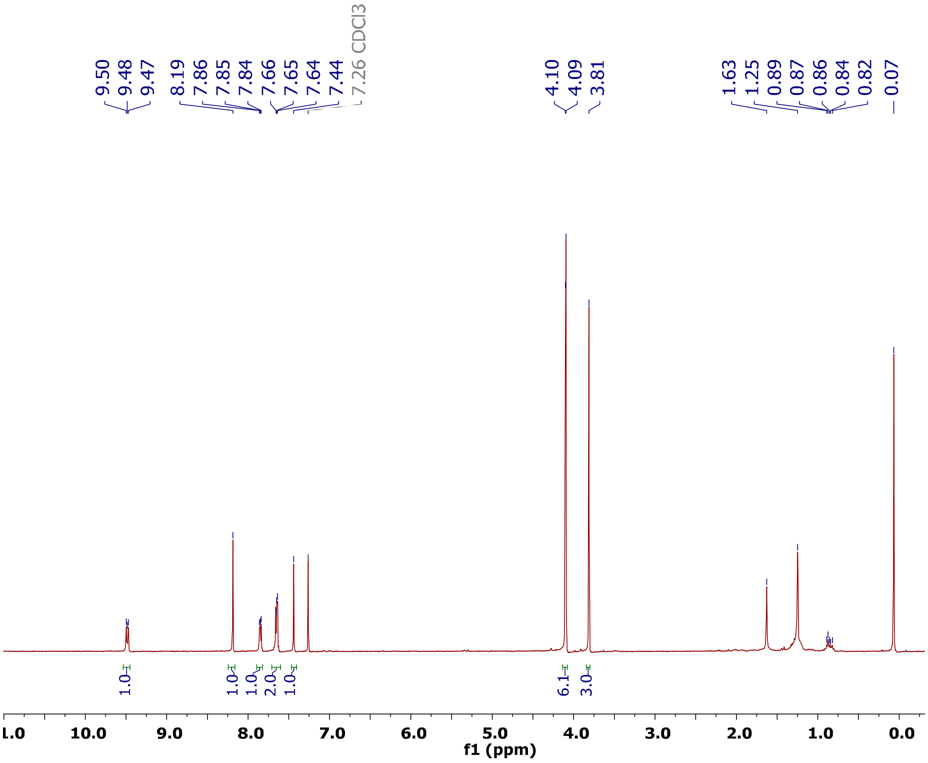

Figure S9. ^{1}H-NMR spectra of cepharadione B (**3**).

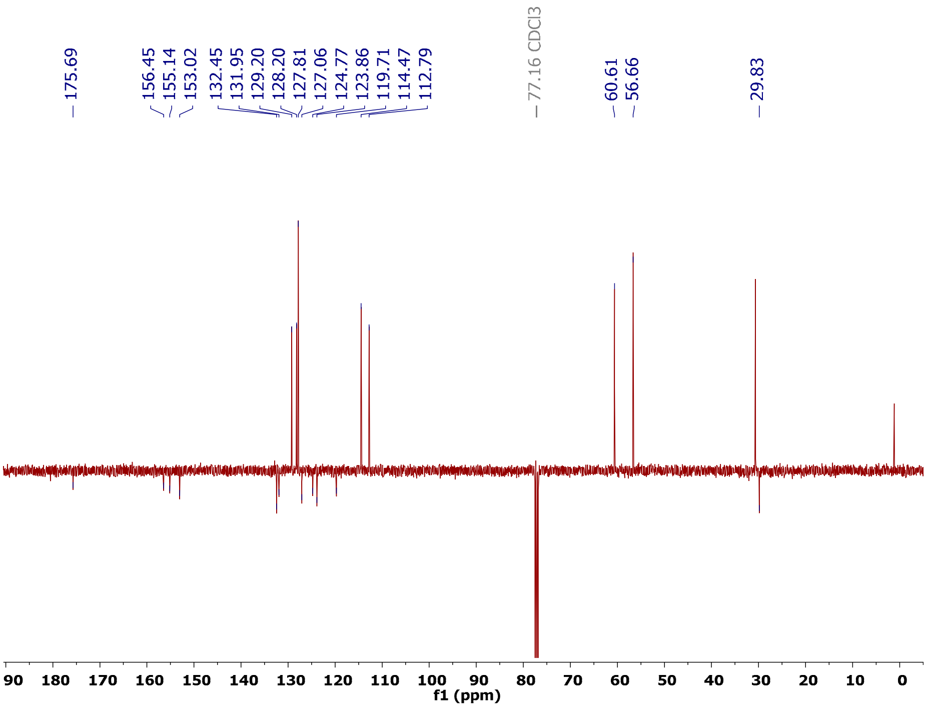

Figure S10. APT spectra of cepharadione B (**3**).

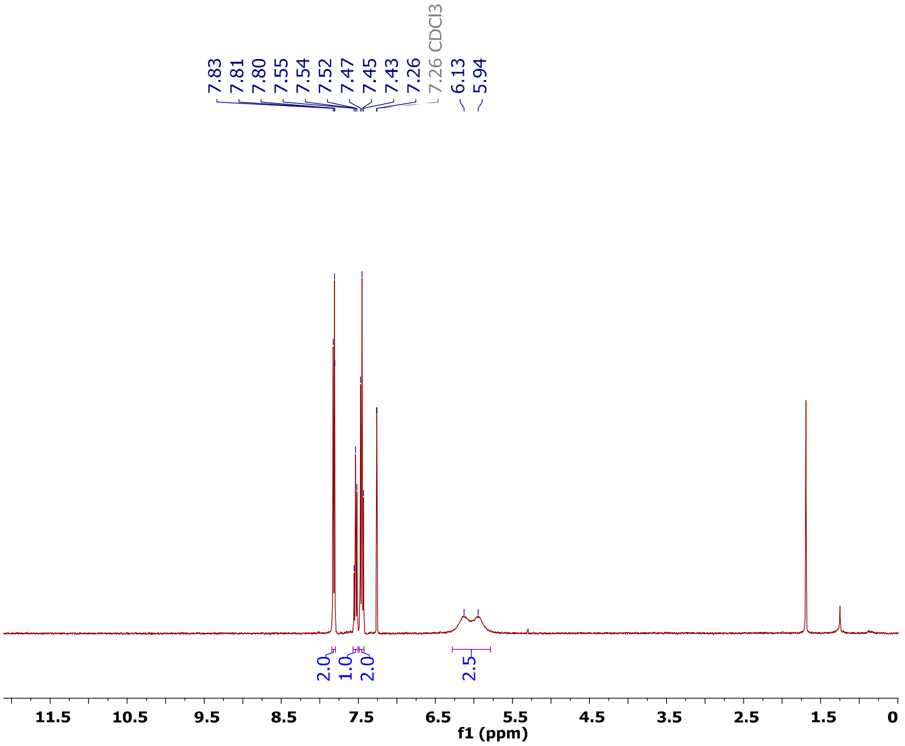

Figure S11. ^{1}H-NMR spectra of benzamide (**4**).

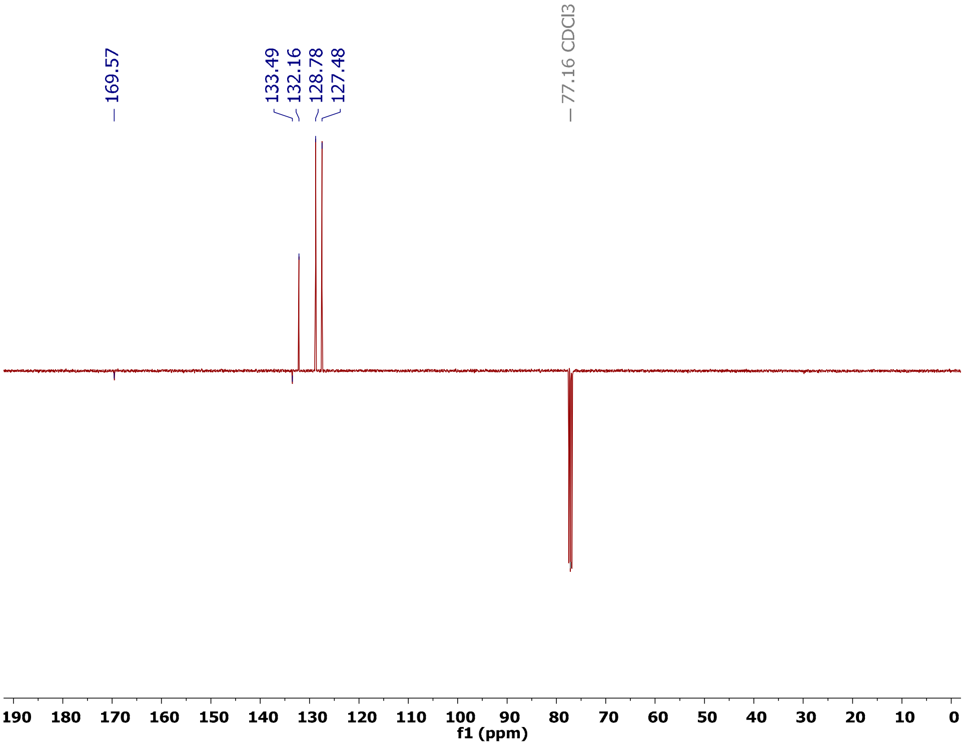

Figure S12. APT spectra of benzamide (**4**).

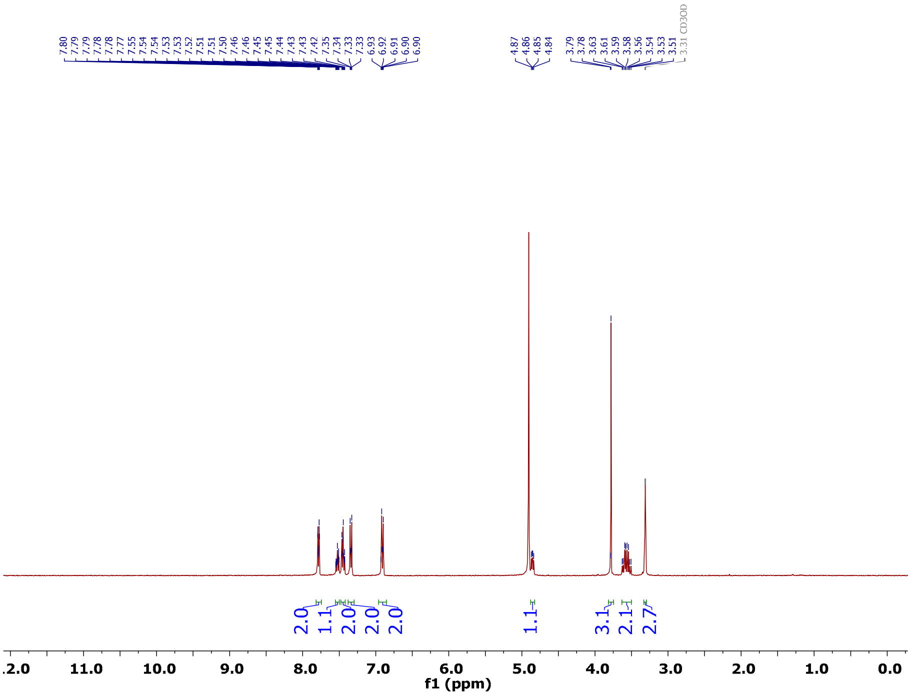

Figure S13. ^{1}H-NMR spectra of tembamide (**5**).

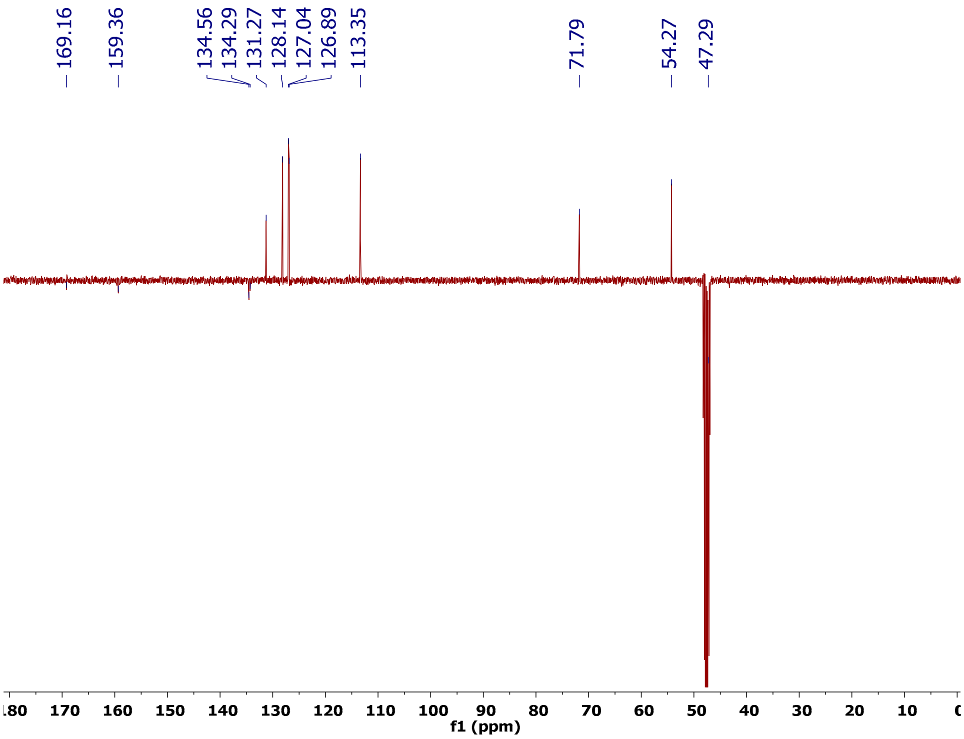

Figure S14. APT spectra of tembamide (**5**).

Table S2. Percentage growth of *P. aeruginosa* on exposure to compounds isolated from *P. pertomentellum*.

**Compound**	**Concentrations µg/mL**			
	**250**	**125**	**62.5**	**31.2**
1	107.5 ± 2.5	95.8 ± 3.4	94.9 ± 5.6	101.1 ± 1.8
**2**	88.7 ± 3.2	88.9 ± 3.6	86.7 ± 0.9	101.3 ± 2.3
**3**	88 ± 1.4	84.5 ± 2.7	87.4 ± 1.5	101.1 ± 1.8
**4**	87.5 ± 7.3	82.9 ± 4.2	84.8 ± 3.7	91 ± 5.7
**5**	91.2 ± 9.9	83.9 ± 3.9	82.4 ± 4.3	91 ± 5.7
**Gentamicine(2 µg/mL)**	2.1 ± 1.3			

Table S3. Percentage biofilm formation and virulence factors production of *P. aeruginosa* on exposure to compounds isolated from *P. pertomentellum*.

**Compound**	**Bioassay**	**Concentrations µg/mL**			
		**250**	**125**	**62.5**	**31.2**
**1**	**Biofilm**	63.3 ± 18.9*	71.8 ± 10.4*	72.9 ± 21.3*	56.4 ± 12.7*
	**Piocianina**	67.1 ± 32.29*	77.9 ± 61.0*	71.2 ± 33.7*	125.2 ± 16.1
	**Proteasas**	81.7 ± 23.7	76.7 ± 27.1*	89.1 ± 21.4	100.3 ± 20.1
	**Elastasas**	49.4 ± 15.7*	48.8 ± 21.9*	55.1 ± 14.7*	66.0 ± 12.1*
**2**	**Biofilm**	93.1 ± 8.3	53.4 ± 3.6*	64.3 ± 10.2*	66.6 ± 4.3*

	**Piocianina**	68.4 ± 14.4*	77.3 ± 12.2	94.2 ± 13.1	110.8 ± 13.1
	**Proteasas**	94.3 ± 8.3	98.9 ± 4.8	83.2 ± 19.8	85.8 ± 20.4
	**Elastasas**	91.9 ± 19.6	66.8 ± 18.1*	60.1 ± 9.9*	62.7 ± 7.4*
3	**Biofilm**	44.2 ± 7.9*	45.4 ± 4.9*	46.7 ± 13.6*	41.5 ± 8.9*
	**Piocianina**	34.6 ± 2.5*	59.3 ± 17.2*	47.9 ± 13.7*	102.1 ± 16.1
	**Proteasas**	77.1 ± 6.1*	86.9 ± 7.1	79.9 ± 15.6	81.1 ± 8.7
	**Elastasas**	58.8 ± 9.2*	80.7 ± 15.3	57.1 ± 12.7*	63.8 ± 11.5*
4	**Biofilm**	135.9± 36.8	102.4 ± 17.7	35.6 ± 4.9*	34.2 ± 3.1*
	**Piocianina**	93.5 ± 9.9	76.9 ± 20.8	79.2 ± 10.6	55.6 ± 14.1*
	**Proteasas**	91.2 ± 12.4	89.1 ± 7.5	89.5 ± 10.6	83.9 ± 6.3
	**Elastasas**	84.8 ± 19.9	74.1 ± 13.3*	69.1 ± 18.4*	68.3 ± 20.1*
5	**Biofilm**	41.2 ± 6.9*	59.1 ± 8.7*	66.1 ± 13.6*	81.9 ± 18.1
	**Piocianina**	71.7 ± 15.4*	78.8 ± 22.6	108.1 ± 12.9	82.1 ± 19.6
	**Proteasas**	81.3 ± 15.8	95.5 ± 11.3	103.9 ± 2.4	95.6 ± 8.5
	**Elastasas**	68.7 ± 19.1*	87.1 ± 15.7	88.2 ± 5.3	82.7 ± 14.1

Data are represented the mean ± standard deviation of five independent replicates. *Indicate a significant difference according to Duncan's test ($p < 0.05$).
